# Omnidirectional, broadband light absorption using large-area, ultrathin lossy metallic film coatings

**DOI:** 10.1038/srep15137

**Published:** 2015-10-09

**Authors:** Zhongyang Li, Edgar Palacios, Serkan Butun, Hasan Kocer, Koray Aydin

**Affiliations:** 1 Department of Electrical Engineering and Computer Science, Northwestern University, Evanston, IL 60208, United States; 2 Department of Electrical Engineering, Turkish Military Academy, Ankara, 06654, Turkey

## Abstract

Resonant absorbers based on nanostructured materials are promising for variety of applications including optical filters, thermophotovoltaics, thermal emitters, and hot-electron collection. One of the significant challenges for such micro/nanoscale featured medium or surface, however, is costly lithographic processes for structural patterning which restricted from industrial production of complex designs. Here, we demonstrate lithography-free, broadband, polarization-independent optical absorbers based on a three-layer ultrathin film composed of subwavelength chromium (Cr) and oxide film coatings. We have measured almost perfect absorption as high as 99.5% across the entire visible regime and beyond (400–800 nm). In addition to near-ideal absorption, our absorbers exhibit omnidirectional independence for incidence angle over ±60 degrees. Broadband absorbers introduced in this study perform better than nanostructured plasmonic absorber counterparts in terms of bandwidth, polarization and angle independence. Improvements of such “blackbody” samples based on uniform thin-film coatings is attributed to extremely low quality factor of asymmetric highly-lossy Fabry-Perot cavities. Such broadband absorber designs are ultrathin compared to carbon nanotube based black materials, and does not require lithographic processes. This demonstration redirects the broadband super absorber design to extreme simplicity, higher performance and cost effective manufacturing convenience for practical industrial production.

Electromagnetic super absorbers have been widely sought across radio frequency (RF), terahertz (THz), Infrared (IR) and optical spectra with significant applications including thermophotovoltaics^1^, photodetections, signature control^2^,^3^,^4^, thermal imaging^5^,^6^ and thermal emission^7^,^8^,^9^. Most recently, plasmonic nanostructures/nanoparticles and metamaterials have been studied extensively and demonstrated as strong candidates for optical super absorbers^1^,^2^,^7^,^10^,^11^,^12^,^13^,^14^. Ideal “blackbody” absorbers are supposed to offer unity absorption over broadband operation range as well as polarization and angular independent spectral response^3^,^15^,^16^. Such absorbers based on nanoscale structures enable great flexibility in achieving desired absorption spectral features by engineering the size, geometry, periodicity of nanostructures. In recent years, there has been wide variety of research activities that push the limits of plasmonic absorbers from narrowband^7^,^11^,^12^,^17^ to broadband^1^,^18^,^19^,^20^ absorption (BBA). Serving as a thermal emitter and coupled with a photovoltaic diode cell, a basic BBA-based thermophotovoltaic system could convert energy from heat to electricity via photons absorption. However, despite the elaborate nanoscale geometric features, there is still large room for further performance improvement in terms of absorption, bandwidth and most importantly omnidirectional absorption characteristics^10^,^21^,^22^,^23^. Furthermore, multiple costly nano-fabrication processing steps and precise manufacturing required to achieve satisfactory absorption functionality^24^,^25^,^26^,^27^,^28^,^29^ turns out to be one of the most critical challenges restricting nanoscale patterned materials from achieving practical applications that require large-area absorbers.

In order to overcome the critical limitation confronted with large-area “blackbody” absorbers, several recent studies^24^,^25^,^26^,^27^,^28^,^29^,^30^,^31^,^32^,^33^,^34^ have reported that utilizing stacked lossy of high-index ultra-thin film layers could offer potential solutions to obtain high absorption at specific wavelength. Specifically, simply coating Ge thin films with thickness of 7–25 nm on flat Au surfaces could exploit strong interference effects and achieve high absorption (80%–97%) at resonant wavelength of visible to near IR region^23^. However, the extension of film coatings design targeting for optical “blackbody” of broadband perfect absorption with omnidirectional features still remains a challenge.

In this paper, we report simple tri-layer Cr/SiO_2_/Cr uniform films on Si substrate that could enable omnidirectional, broadband response with near-ideal absorption and polarization-independence across the entire visible spectrum. Measurements of the optimized stack coatings show strong agreement with FDTD simulations and TMM model calculations, demonstrating near-unity absorption as high as 99.58% with average absorption of 97.07% from 450 nm to 800 nm. The flat-band absorption is not only completely independent of light polarization but also exhibits angle-independent absorption behavior for oblique incidences up to ±60 degree. Such thin film coatings represent a significant step towards realization of high-performance visible or IR planar “blackbody” allowing extremely simple BBA designs practical for industrial production.

## Results

**Figure 1:**
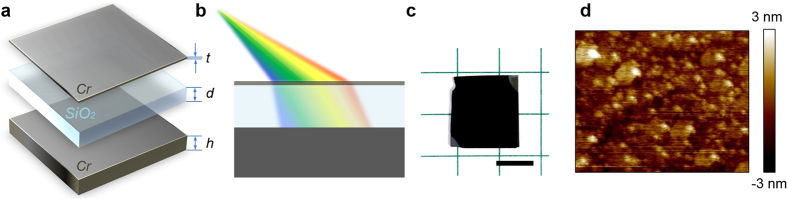
Schematic configurations and performance of the broadband absorption cavity. (**a**) Schematic diagram of the Cr-SiO_2_-Cr tri-layer thin film stack. The geometrical parameters are: *t* = 3 nm, *d* = 95 nm and *h* = 100 nm. (**b**) Schematic of perfect absorption for oblique broadband illumination of visible light. (**c**) Optical image of CrOCr sample, revealing totally black color in contrast with background notebook. The length of scale bar is 1 cm. (**d**) AFM image on top of CrOCr sample.

A schematic illustration of Cr/SiO_2_/Cr (CrOCr) film coatings for BBA is displayed in Fig. 1a. We consider the geometric thickness to be *t* = 3 nm, *d* = 90 ~ 100 nm and *h* = 100 nm for the top thin Cr coating, SiO_2_ core layer and Cr backside reflector, respectively. With this configuration, illustrated in Fig. 1b, we targeted and successfully achieved polarization-independent BBA across entire visible range with near-ideal absorption of greater than 98% over a large oblique angular tolerance of ±60 degree. For this work, different metallic, semi-metallic, lossy and high-index semiconductor materials including Ag, Au, Al, Cu, Cr, In, Si, Ge and GaAs were used in our simulations for constructing the high energy conversion cavity (see Supporting Information). In order to enable perfect broadband absorption, absorption was eventually optimized using Cr to serve as ground plane and top thin-film coating owing to its optically lossy nature and less dispersive characteristics. SiO_2_ was selected for its high transparency broadband window with non-dispersive optical properties over the visible wavelength range. An optical image of a fabricated CrOCr sample (*t* = 3 nm, *d* = 95 nm and *h *= 100 nm) is displayed in Fig. 1c that looks completely black since omnidirectional incident light in visible range is completely absorbed. Such CrOCr films are coated on a polished silicon wafer substrate in our work. The Atomic Force Microscopy (AFM) image is shown in Fig. 1d with surface roughness parameters Rq = 0.837 nm and Ra = 0.661 nm and Rmax = 6.52 nm. Such surface roughness are induced by the film depositions of three layers on Si wafer, especially the SiO_2_ layer could induce the major surface differences. However, the surface roughness could enhance the absorptive characteristics of the CrOCr sample.

**Figure 2:**
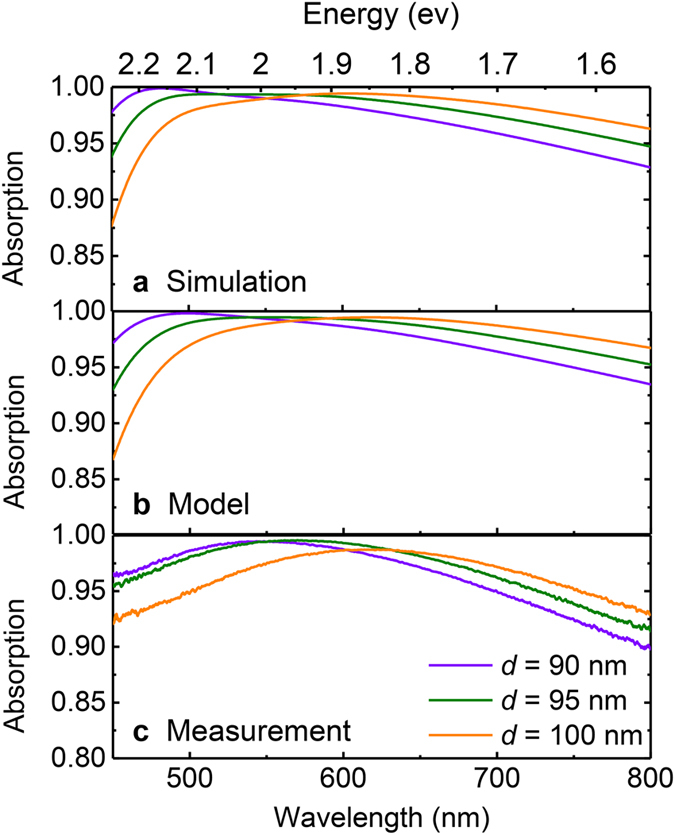
Performance of perfect broadband absorption for normal illumination. (**a)** Simulated, (**b**) modeled and (**c**) measured absorption spectra for CrOCr cavity with different dielectric thickness *d* = 90, 95 and 100 nm.

The spectral characteristics of the CrOCr coatings are calculated by performing electromagnetic full wave Finite-Difference Time-Domain (FDTD) method using commercial software package Lumerical™. With respect to the normal incidence simulation, a planar source is normally incident to the top surface of CrOCr coating with arbitrary polarization and absorption spectrum is retrieved from scattering parameters by *A* = 1–*R*–*T* = 1–*R*, where *A*, *R* and *T* is the absorption, reflection and transmission (the bottom Cr layer is optically thick so as to minimize *T* to be zero). The optical influence from Si substrate could be neglected since all the light transmission is blocked by the bottom thick Cr film. The simulated normal incidence spectra for three different thickness (*d* = 90 nm, 95 nm and 100 nm) of SiO_2_ are shown in Fig. 2a. It is shown that for an oxide thickness of *d* = 95 nm, near-perfect absorption is achieved with maximum absorption value exceeding 99.36% and an average value equal to 97.89% across the visible range from 450 nm to 800 nm. For cases where *d* = 90 or 100 nm, the BBA band is slightly shifted, but maintains its high absorption features which demonstrate the structural robustness and insensitivity to slight variation of the oxide geometry. Basically, the absorption band is produced by a single wide and flat-band resonance located around 500–600 nm with extremely high absorption and engineered low quality factor of cavity construction. In addition to FDTD simulations, Transfer-Matrix Method (TMM) was also utilized to reproduce the BBA effect, which has been extensively discussed in Experimental Section. As exhibited in Fig. 2b, the absorption retrieved from TMM model also predicts very similar spectral trend over 450 nm–800 nm, whose resonance centered around 560 nm with maximum and average absorption of 99.47% and 98.14%.

To verify the numerical FDTD simulation and TMM model calculation, thin-film samples were produced by depositing the tri-layer Cr/SiO_2_/Cr films on a polished silicon wafer using Electron Beam (E-beam) Evaporator. Three different SiO_2_ thickness variations were realized by etching the same oxide thickness coatings for different times by Reactive-Ion Etching (RIE). Measurements of the three CrOCr samples have shown strong agreement with FDTD simulations and TMM model calculations in terms of the overall shape, bandwidth and spectral position, as plotted in Fig. 2c. For the case where *d* = 95 nm, the experimental measurement exhibits near-unity absorption as high as 99.58% at 572 nm with average absorption of 97.07% from 450 nm to 800 nm. Even for an ultra-wide wavelength range from 300 nm to 900 nm, which includes UV up to the near-IR regime, the measured absorption is no less than 90%. For other thickness cases where *d* = 90 nm or 100 nm, the flat-band absorption is respectively blue-shifting and red-shifting slightly but still maintaining the broadband absorption characteristics in the visible frequency regime, as shown in Fig. 2. Therefore, the composition of ultra-thin Cr coupling with an optically thick Cr film separated by a transparent spacer yields almost perfect absorption behavior, not only at specific wavelength, but across the entire visible regime and beyond.

**Figure 3:**
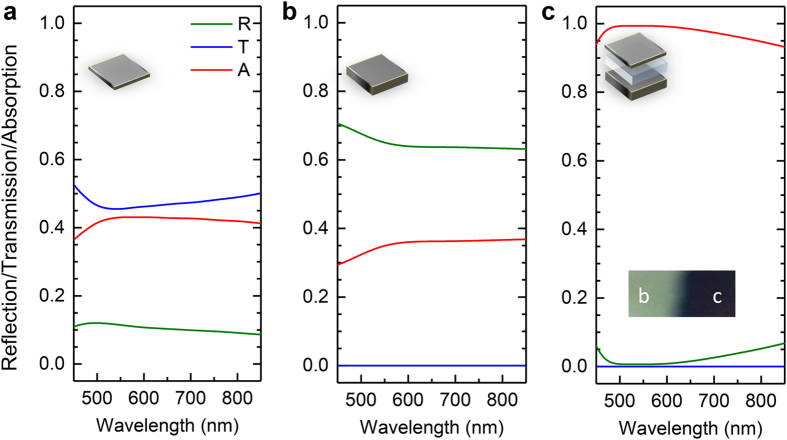
Reflection, Transmission and Absorption spectral comparison for differentiated Cr layer and integrated CrOCr cavity. Simulated spectra for (**a**) 3 nm thick Cr layer, (**b**) 100 nm thick Cr layer, and (**c**) the combined CrOCr stack. The inset is the surface optical image for samples in (**b**,**c)**.

Despite design conciseness, integrating tri-layer films is of great significance in order to maintain the near-unity absorption without sacrificing the flat broadband feature. By analyzing the spectral response of CrOCr cavity layers individually, we elucidate the respective role of each layer. The reflection *R*, transmission *T* and absorption *A* spectra for ultra-thin layer (3 nm thickness) of Cr and thick substrate (100 nm thickness) Cr film are respectively drawn in Fig. 3a,b. The 3 nm-thickness Cr layer reveals high transparency of ~50% transmission with ~10% reflection. On the other hand, thick Cr film could block the broadband light transmission but encounters over 60% light reflection. In both cases, the ultimate calculated absorption does not exceed 50%, which illustrates that the extraordinary BBA feature by CrOCr coating is unachievable from individual lossy films. Only when coupling thin and thick Cr layers separated by a dielectric spacer is it possible to enhance the best of BBA features as displayed in Fig. 3c. The inset figure of Fig. 3c compares the surface images which reveal colors from the bulk Cr substrate, without top Cr coating and the CrOCr coating. The half-black and half-grey sample is fabricated by covering half of the sample with the shutter during E-beam evaporation, thus coating one half with oxide and top Cr thin film while leaving the other half with only the bottom Cr layer. The gradual color variation from grey to totally black confirms again the satisfactory performance of broadband, near-ideal absorption and also proves the conciseness of design and feasibility for actual production.

**Figure 4:**
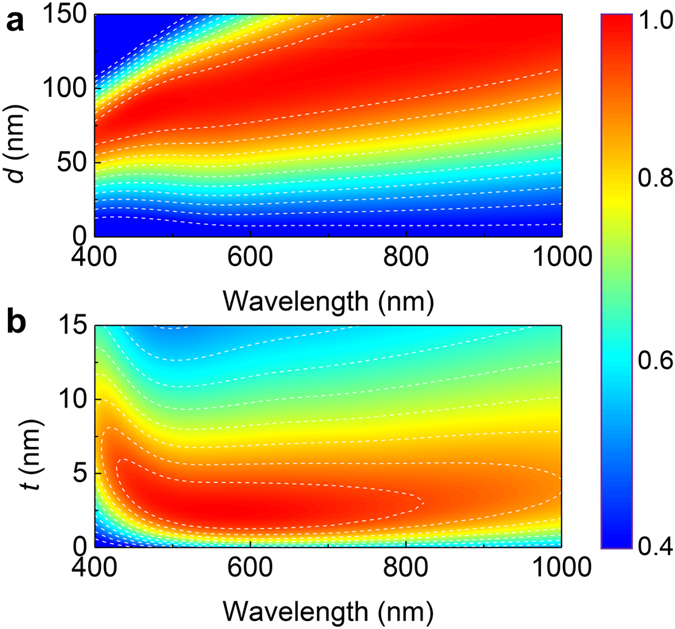
Absorption dependence on the geometry of planar CrOCr coating. (**a**) Absorption contour plotted as a function of wavelength and dielectric thickness *d*. (*t* = 3 nm and *h* = 100 nm) (**b)** Absorption contour plotted as a function of wavelength and top Cr thickness *t*. (*d* = 95 nm and *h* = 100 nm)

As a means for optimizing the proposed design to an ideal optical absorber, it is instructive to study the layered geometry for CrOCr cavity. We performed numerical FDTD simulations to estimate the spectral evolution as a function of SiO_2_ thickness (*d*) and top Cr thickness (*t*). The core thickness *d* of the dielectric layer dictates the center wavelength of resonant absorption, as shown in Fig. 4a. As increasing *d*, the absorption band considerably redshifts while broadening. For highest total absorption over the visible regime, we design the dielectric layer with thickness around 90–100 nm. In terms of top Cr thickness *t*, the optimum value for maximized absorption turns out to be *t* = 2–4 nm as exhibited in Fig. 4b. Essentially, the geometric size of the very thin Cr layer is critically responsible for balancing the trade-off between light penetration and cavity absorption. A thicker top Cr film tends to reflect back the light illumination from the cavity, whereas a thinner film of Cr is not thick enough to have high losses required for high absorption. For the case of *t* = 2–4 nm, we are thus able to boost the average absorption by optimizing the interplay between cavity confinement and the lossy nature of thin films.

**Figure 5:**
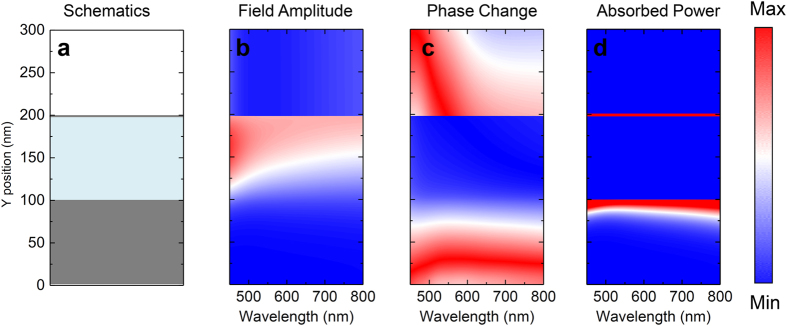
Field magnitude and phase change as well as absorber power distributions along propagation position as a function of wavelength. (**a**) The schematics of CrOCr stack configuration. The triple-layer along y-axis is respectively, Cr (0–100 nm), SiO2 (100–195 nm) and Cr (195–198 nm). (**b**) Simulated electric field amplitude, (**c**) phase change, and (**d**) absorbed power along *y*-axis as a function of wavelength.

The simple CrOCr coating resembles an asymmetric Fabry-Perot-type (FP-type) nanocavity composed of a lossless dielectric core with a top partially transparent/reflective lossy layer and backside reflector as schematically shown in Fig. 5a^26^,^27^,^28^,^29^. Nevertheless, it is essentially distinctive from conventional FP-type resonator in two aspects. First, conventional FP-type resonator usually exhibits highly wavelength selective feature in transmission or reflection spectra, but the spectral modulation is free from any energy conversion/absorption process. In contrast, by incorporating a high-loss metallic boundary into cavity construction, the CrOCr cavity could yield extraordinary energy conversion efficiency as a perfect absorber. Second, instead of operation at discrete spectral region, the CrOCr could exceed the limitation of narrow-band spectral behavior and function over the entire visible spectrum. The electric field profile (Fig. 5b), field phase (Fig. 5c) and absorbed power (Fig. 5d) distributions over the cross-section could illustrate the BBA schematics. E-fields are shown to be highly confined by boundary reflective metals and trapped in the dielectric layer not only at resonance wavelength but across the entire visible range, as depicted in Fig. 5b. The variation of trapping schemes for various wavelengths is due to the different penetration lengths into the cavity as well as material dispersion. Figure 5c displays the uniformity of phase change for the trapped wave inside the dielectric layer, which confirms the broadband light trapping and absorption mechanism. From the calculated absorbed power distribution (Fig. 5d), the high efficiency of absorption is attributed to both the top and bottom Cr layers. Nevertheless, the most absorbed power occurs in the top Cr layer, accounting for more than 15 times the dissipation inside the bottom Cr.

**Figure 6:**
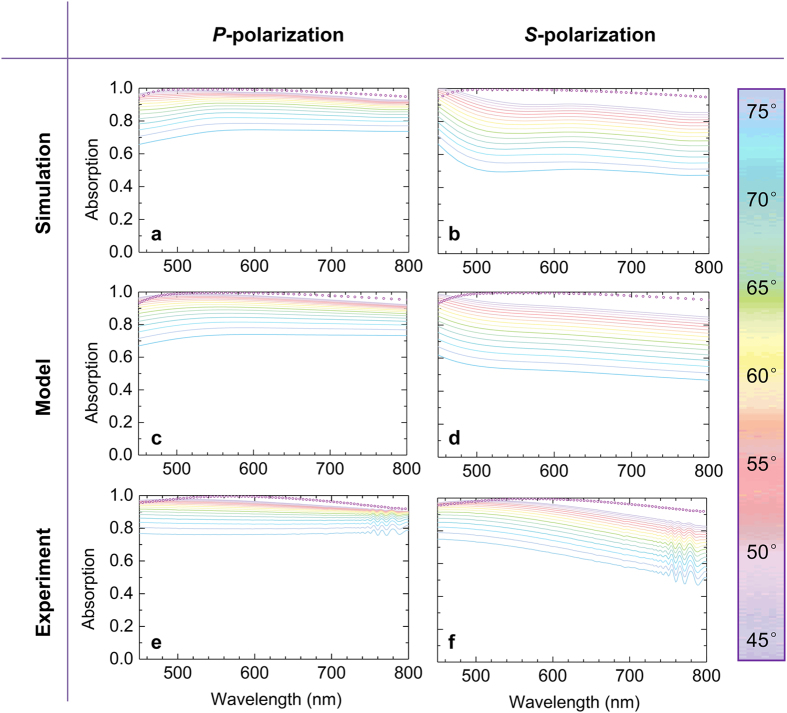
Angle and polarization dependent absorption spectra for CrOCr sample. (**a,b)** Simulated, (**c,d**) modeled, and (**e,f**) experimental spectra for different oblique incidence angles from 45 degree to 75 degree. (**a**,**c**,**e)** are for *P*-polarization cases. (**b**,**d**,**f)** are for *S*-polarization cases. The dots data are normal incidence spectra.

The CrOCr cavity not only could exhibit broadband absorption behavior for normal incidence light, but is also insensitive to the incident angle. Since the thickness of each stacked thin layer is sub-wavelength, the phase change accumulation is not considerable enough compared to the normal incidence case and hence the schematics could also work for wide oblique incidence. As shown in Fig. 6a–f by numerical simulations, TMM modeling and experimental measurement, the angle-dependence spectra demonstrates that super broadband absorption is still retained for large incidence angles. For normal incidence, the planar cavity is polarization-independent, whereas for oblique incidence, *S*-/*P*-polarization could exhibit different absorption behavior. For *P*-polarized light with a 45 degrees incidence angle, the measured spectrum still displays extreme high and flat broadband absorption with maximum and average absorption of 97.8% and 95.8%, respectively. As shown in Fig. 6e, the spectrum from 45 degrees nearly overlaps the normal incidence case, which demonstrates the omnidirectional broadband absorption. Even with a 60 degrees angle of incidence, the BBA uniformly reduces only ~ 4% of average absorption. When increasing the incidence angle to 75 degrees, the nearly flat-band absorption tends to also decrease slightly further. For *S*-polarization, when increasing the oblique angles spectral evolution exhibits similar modulation with P-polarization but with larger reduction of absorption in the longer wavelength range.

## Discussion

In this study we propose a broadband, almost perfect absorber that is insensitive to angle and polarization across the entire visible range. The absorber is based on a simple, lithography free design consisting of tri-layer subwavelength thick Cr and oxide films. The maximum absorption from 450 nm to 800 nm is 99.58% and the corresponding average absorption is 97.07% for the normal incidence case. Even for an ultra-wide wavelength range from 300 nm to 900 nm including UV, the measured average absorption is no less than 90%. Extensive characterization by TMM modeling, numerical simulation and experimental measurement, show that such a “blackbody” response is demonstrated to retain its high and flat absorption performance over a wide incidence angle range of  ±60 degrees for oblique incidence cases. By individually examining the layers in integrated CrOCr stack, the spectra for each film medium was calculated to reveal the function of each stacked layer and to emphasize the extraordinary spectral features arising from such simple tri-layered stacks. Essentially, the BBA effect is attributed to extremely low quality factor engineering of high loss asymmetric Fabry-Perot-type cavities. Within such lossy cavities, the E-field is highly confined by boundary reflective metals and trapped in the dielectric layer, not only at resonance wavelength, but across the entire visible range. This lithography-free approach based on thin film coatings capable of achieving omnidirectional BBA behavior significantly improves the performance and allows for easier manufacturing in order to produce cost effective, robust and practical devices.

## Methods

### Fabrication

In terms of fabrication of the BBA samples, we coated the bottom Cr layers of ~100 nm on polished silicon wafer using electron beam (E-beam) evaporator. Then the SiO_2_ layer of 100 nm thickness was evaporated onto the bottom Cr layer. The other two different oxide thicknesses of 95 nm and 90 nm are processed by Reactive Ion Etching (RIE) facility to etch the 100 nm-thickness SiO_2_ for 10 seconds and 20 seconds, respectively. The actual etching rate is ~33 nm per minute. Finally, we coated the top 3 nm thick top Ag layers for the 3 samples together. In addition to the three Cr-SiO_2_-Cr layers, we also coated on sample surfaces with ~3 nm SiO_2_ layer for the sake of prevention top Cr surface from oxidation.

### Optical Measurement

A microscope equipped with a spectrometer consisting of a 303-mm-focal- length monochromator and Andor Newton electron multiplication charge-coupled device (EM-CCD) camera was utilized for optical characterization. Broad band illumination is generated by a broadband halogen lamp and a linear polarizer was inserted into the light pathway to polarize the incident light. Reflected and transmitted light was collected using a 2X Nikon microscope objective with a numerical aperture of 0.06. For calibration of reflection, we first measured the reflection from a broadband dielectric mirror (Edmund Optics #64–114) with an average reflection of 99% between 350 and 1100 nm. Measured reflection from CrOCr samples was then calibrated using the reflection spectra of the dielectric mirror. The oblique incidence measurement is taken using Spectroscopic Ellipsometer (Keck-II) to obtain angular reflection spectra from 45 degree to 75 degree with step size of one degree.

### Transfer Matrix Method (TMM)

Regarding TMM modeling, we assume that a plane wave source normally incident and propagate along z-axis through the stack of Cr/SiO_2_ layers, the field within each layer could be treated as superposition of forward-traveling (transmitted) and backward-traveling (reflected) wave with wave number *k* and a transfer matrix could represent the propagation through interface or within medium. According to TMM, it can be described as


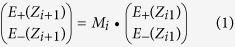


where *M*_i_ can be determined by material parameters. By cascading the transfer matrix for each layer, the whole system transfer matrix can be obtained from which one could derive the transmission *T* and reflection *R* of the film coating.

### FDTD simulation

Full-field electromagnetic wave calculations were performed using Lumerical, a commercially available finite-difference time-domain (FDTD) simulation software package. Simulations for the planar films were performed in 2D layout. A unit cell of 200 nm along the *x*-axis and 2000 nm along the *y*-axis was selected for the planar structure and was simulated using periodic boundary conditions along the *x*-axis and perfectly matched layers (PML) along the propagation of electromagnetic waves (*y*-axis). Plane waves were launched incident to the unit cell along the +*y* direction, and reflection is collected with a power monitor placed behind the radiation source; transmission is collected with a power monitor placed behind the structure. The time step is set to be with dt stability factor of 0.99 and dt of 0.000818564 fs. The simulation time is 10000 fs and the convergence criteria is set to be with auto shutoff min of e^−5^. The mesh size is set to be 2 nm along *x*-axis and 0.2 nm along *y*-axis. The coefficients number for Cr fitting is 7 with RMS error of the approximation to be 0.328868. Electric field and phase change as well as absorber power distribution cross-section are detected by a 2D field profile monitors in x-y plane. The complex refractive index of Cr, SiO_2_ and other material data for simulation are all utilized from the data of Palik^35^.

## Additional Information

**How to cite this article**: Li, Z. *et al*. Omnidirectional, broadband light absorption using large-area, ultrathin lossy metallic film coatings. *Sci. Rep*. **5**, 15137; doi: 10.1038/srep15137 (2015).

## Supplementary Material

Supplementary Information
