# Supplementary material for: Omnidirectional, broadband light absorption using large-area, ultrathin lossy metallic film coatings
Source: Sci Rep. 2015 Oct 9;5:15137. doi: 10.1038/srep15137 (PMC4598830; doi:10.1038/srep15137)
Supplement: Supplementary Information [file srep15137-s1.pdf]

# Supplementary Information

# Omnidirectional, broadband light absorption using large-area, ultrathin lossy metallic film coatings

**Zhongyang Li[1], Edgar Palacios[1], Serkan Butun[1], Hasan Kocer[2], & Koray Aydin[1*]**

[1]Department of Electrical Engineering and Computer Science, Northwestern University, Evanston, IL 60208, United States
[2]Department of Electrical Engineering, Turkish Military Academy, Ankara, 06654, Turkey
*E-mail: aydin@northwestern.edu

## Boundary material comparison for metal-oxide-metal film coatings

For this work, we have demonstrated that utilizing highly lossy material Cr as boundary layers for the triple-layer film coating enables BBA spectral feature. The geometry of the MIM design has been optimized for Cr, which makes a good match between the effective impedance of the air and the CrOCr medium. In such way, the reflected light could be minimized for the mode matching condition. To gain more insight into the impact of material property of the confinement layer on the thin-film coating's absorption performance, it is necessary to investigate that different material serve as the boundary film layers. Changing the material of the triple-layer films with the same geometry will totally change the optical properties. Here different metallic, semi-metallic and lossy/high-index semiconductor materials including Ag, Au, Al, Cu, Cr, In, Si, Ge and GaAs were trialed as top and bottom layers for constructing the

high energy conversion cavity. Owing to the distinctive optical properties in visible domain between materials, the simulated absorption spectra demonstrate various spectral characteristics in absorption, as shown in **Figure S1**. Among all the absorption spectra for different materials, only Cr could achieve near-unity absorption with flat broadband feature. That is because Cr possesses much higher real part of refractive index (n) which builds stronger cavity confinement. In addition, for ideal absorption purpose, Cr enjoys much larger imaginary part of index (k) than other semiconductors and metals, thus giving rise to near-unity absorption over the entire visible regime.

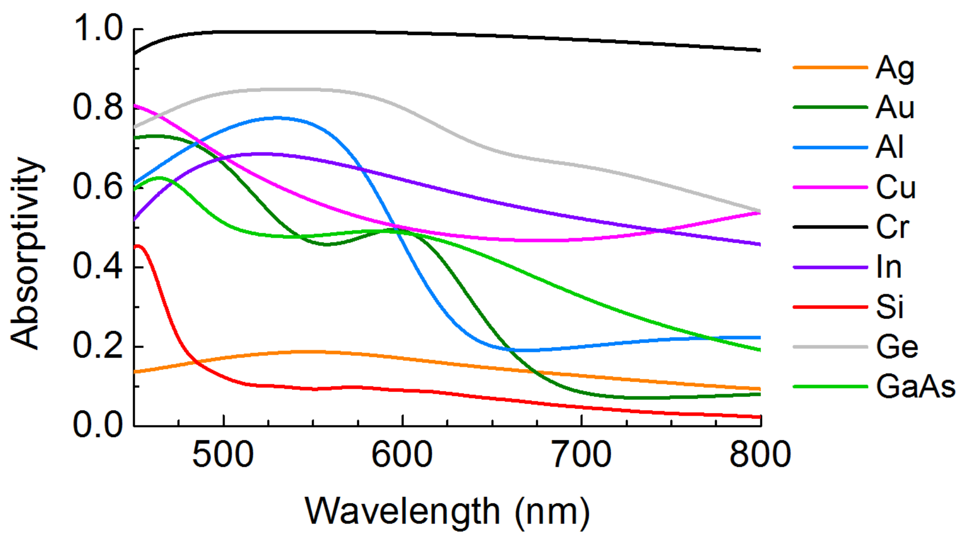


**Figure S1.** Simulated absorption spectra for metal-oxide-metal film coatings with different material serving as boundary layers.

## <u>Surface roughness impact of the CrOCr sample on absorptive characteristics</u>

We have performed the FDTD simulations including the surface roughness from experimental parameters and the spectra are shown as below **Figure S2**. Both smooth and rough surface could exhibit BBA characteristics. It is worth noting that the achieved performance of BBA by thin film of Cr is not due to the surface roughness. Regarding the ideal simulation results which matched well with the measurements, we utilized the ideal smooth metallic and insulator layers for the MIM structure and the absorption spectrum is of quite broad and high absorption in visible range. Such simple design for absorption enhancement does not depend on any surface roughness effect and is also essentially distinctive from nanostructured absorbers, where the BBA performance is due to the complex plasmonic resonances.

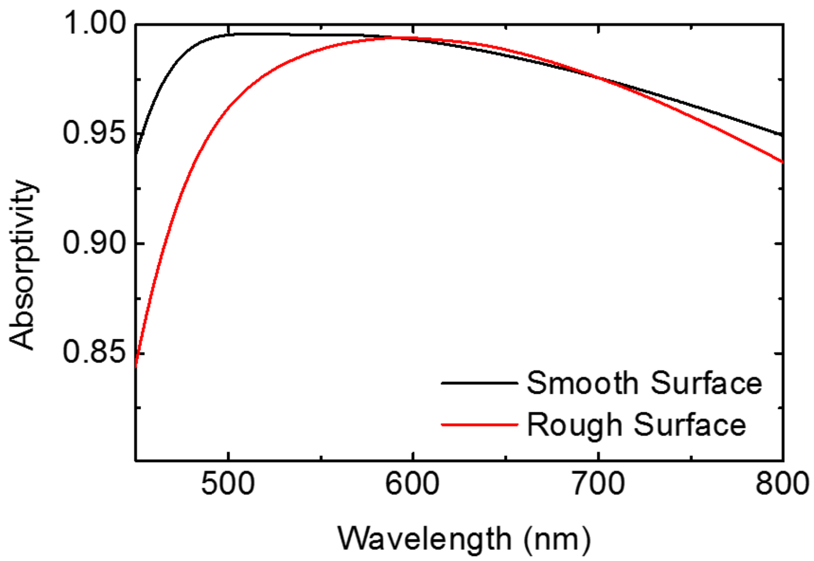


**Figure S2**. Simulated spectra of smooth and rough surface for CrOCr design
